# Combination of trichosanthes cucumerina L. compounds: an analysis for novel effects of anticancer cell activites as probes for pharmacological studies

**DOI:** 10.1186/2051-1426-1-S1-P134

**Published:** 2013-11-07

**Authors:** Md Ariful Haque Mollik

**Affiliations:** 1 Biological Sciences, Peoples Integrated Alliance, Bogra Sadar, Bangladesh; 2 Research and Development, Prescience Trust Funds, Phoenixville, PA, USA

## 

Prostate cancer is a hormone-dependent cancer and its proliferation is stimulated by endogenous steroid hormones. Aromatase (CYP19) and 5α-reductase (SRD5A) are the key enzymes that synthesize these hormones. Aromatase converts androgens to estrogens and 5α-reductase converts testosterone to dihydrotestosterone. Natural compounds present in the Trichosanthes cucumerina L. delay prostate cancer progression. These compounds may act as inhibitors of steroidogenesis. Trichosanthes cucumerina L. compounds (punicic-, quinic-, gallic-, trans-vaccenic-, and cis-vaccenic-, pelargonidin-, cyanidin-, malvidin-, and delphinidin chloride, epicatechin gallate, epicatechin, kaempferol, and epigallocatechin) were tested in vitro in a hormone-dependent prostate cancer (LNCaP cells) and steroidogenesis (human adrenocortical H295R cells) model. Cells (5000 cells/ml) in 96 well culture plates were exposed to various concentrations of Trichosanthes cucumerina L. compounds for 6 days with a fresh re-exposure after 48 hours. For cytotoxic effects, H295R were exposed once to Trichosanthes cucumerina L. compounds for 24 hours. Cytotoxicity and antiproliferative effects were measured with WST-1 reduction assay. Catalytic activity of CYP19 was determined in H295R cells by tritiated water-release assay. Punicic acid had an antiproliferative effect in LNCaP cells reducing cell growth by 78, 88, and 91% at 30, 45, and 100 μM respectively. Kaempferol reduced proliferation by 30, 48, and 69% at 15, 30, and 100 μM respectively. Punicic acid was not cytotoxic in H295R cells, and at 30 μM, decreased aromatase activity by 75% compared to control. CYP19 was not express in LNCaP cells. Preliminary consequences show that various natural compounds found in the Trichosanthes cucumerina L. have an antiproliferative effects in LNCaP cells and punicic acid acts as an aromatase inhibitor in H295R cells.

